# Stereotactic Body Radiotherapy (SBRT) of Pancreatic Cancer—A Critical Review and Practical Consideration

**DOI:** 10.3390/biomedicines10102480

**Published:** 2022-10-04

**Authors:** Petr Burkoň, Jan Trna, Marek Slávik, Radim Němeček, Tomáš Kazda, Petr Pospíšil, Milan Dastych, Michal Eid, Ivo Novotný, Tomáš Procházka, Miroslav Vrzal

**Affiliations:** 1 Department of Radiation Oncology, Masaryk Memorial Cancer Institute, Zluty Kopec 7, 656 57 Brno, Czech Republic; 2 Faculty of Medicine, Masaryk University, Kamenice 753/5, 625 00 Brno, Czech Republic; 3 Department of Gastroenterology and Digestive Endoscopy, Masaryk Memorial Cancer Institute, Zluty Kopec 7, 656 53 Brno, Czech Republic; 4 Department of Comprehensive Cancer Care, Masaryk Memorial Cancer Institute, Zluty Kopec 7, 656 53 Brno, Czech Republic; 5 Department of Gastroenterology, University Hospital Brno, Jihlavska 340/20, 625 00 Brno, Czech Republic; 6 Department of Hematology, Oncology and Internal Medicine, University Hospital Brno, Jihlavska 340/20, 625 00 Brno, Czech Republic

**Keywords:** pancreatic neoplasms, neoadjuvant therapy, stereotactic body radiotherapy, review

## Abstract

Pancreatic cancer is the third leading cause of cancer death in the developed world and is predicted to become the second by 2030. A cure may be achieved only with surgical resection of an early diagnosed disease. Surgery for more advanced disease is challenging and can be contraindicated for many reasons. Neoadjuvant therapy may improve the probability of achieving R0 resection. It consists of systemic treatment followed by radiation therapy applied concurrently or sequentially with cytostatics. A novel approach to irradiation, stereotactic body radiotherapy (SBRT), has the potential to improve treatment results. SBRT can deliver higher doses of radiation to the tumor in only a few treatment fractions. It has attracted significant interest for pancreatic cancer patients, as it is completed quickly, requires less time away from full-dose chemotherapy, and is well-tolerated than conventional radiotherapy. In this review, we aim to provide the reader with a basic overview of current evidence for SBRT indications in the treatment of pancreatic tumors. In the second part of the review, we focus on practical information with respect to SBRT treatment plan preparation the performance of such therapy. Finally, we discuss future directions related to the use of magnetic resonance linear accelerators.

## 1. Introduction

Pancreatic cancer (PC) is one of the most aggressive cancers, with a 5-year overall survival rate [1] of only around 6% and a median overall survival [2] of up to 13.6 months. This disease is the third leading cause of cancer death in the developed world and is predicted to become the second most common cause of cancer mortality by 2030 [3,4].

Currently, a cure can be achieved only with surgical resection of early diagnosed disease [5,6]. However, patients with borderline resectable/potentially resectable (BRPC) or locally advanced (LAPC) pancreatic cancer have tumor involvement in critical abdominal vessels, which may make an operation procedure challenging or even impossible [7,8]. Surgery may also be contraindicated due to aggressive features, such as elevated CA19-9, or due to other medical comorbidities [9]. In some such cases, neoadjuvant therapy may improve the probability of achieving R0 resection. Moreover, a disease that progresses through preoperative therapy can identify patients with tumor biology that would ultimately be unfavorable for resection [10].

Neoadjuvant therapy often consists of systemic treatment followed by radiation therapy applied concurrently or sequentially with cytostatics [11,12]. As initial therapy for borderline resectable or locally advanced disease, traditionally combined chemotherapy regimens, such as FOLFIRINOX (5-fluorouracil + leucovorin + irinotecan + oxaliplatin) or gemcitabine plus or minus nab-paclitaxel, have been preferably used [13,14,15]. Careful re-evaluation of response with the use of CT and CA 19-9 levels, as well as monitoring of patient performance status and symptoms is required every 2–3 months. Systemic neoadjuvant therapy is recommended for a total period of 3–4 months to downsize the disease and exclude patients with rapidly progressive disease. After confirmation of tumor response or at least disease control, techniques focusing on increasing the local control should be considered. External beam radiotherapy delivered daily for 5–6 weeks by three-dimensional (3D) conformal or intensity-modulated radiation therapy (IMRT) is still the most common treatment course [8]. The limited ability of these 3D techniques to avoid bowel structures and the need to use large treatment fields to cover the pancreas and surrounding nodal areas result in high toxicity rates. Moreover, conventionally fractionated doses of 40 to 60 Grays (Gy), which are based on the tolerability of large-field radiation to the stomach and duodenum, have been shown to have minimal to no impact on the overall survival of patients [16].

Mixed results were reported in phase III randomized trials [2,16,17] evaluating the role of standard doses of radiation delivered with concurrent chemotherapy to chemotherapy alone for the treatment of LAPC. A recent study comparing the neoadjuvant administration of gemcitabine and erlotinib with and without concomitant radiotherapy (50.4 Gy/28 fractions + capecitabine) showed that conventionally fractionated chemoradiation up to 60 Gy can lead to a modest local control benefit but only minimal or no effect on survival [2]. The reason why the benefit to local control is not translated into a benefit in terms of survival is probably multifactorial and largely influenced by the high frequency of metastases observed in this disease. Another possibility is that local control gains were not significant enough to cause a difference in survival, at least in a subset of patients with predominantly locoregional disease progression. This requires an effort to increase the radiation dose.

The recent success of more aggressive and effective chemotherapeutic regimens, such as FOLFIRINOX and gemcitabine plus nab-paclitaxel, has prompted a review of local therapy, which represents an unmet clinical need [13,14]. With improved systemic control, local progression can become a more serious problem in terms of survival and quality of life. However, the local control rate of standard external radiotherapy was disappointing, with an annual rate of local progression of around 50%, leading most academic centers to change their preference from standard dose chemoradiotherapy to new radiotherapy techniques targeting a primary tumor with a minimal margin, i.e., stereotactic body radiotherapy (SBRT) [18].

This shift goes hand in hand with technological developments that enable the delivery of a highly focused radiation dose to the tumor while sparing the adjacent at-risk organs, especially gastrointestinal (GI) structures. These methods include most of the following: targeting radiation beams to fiducials or markers inserted into the tumor [19], daily use of a computed tomography (CT) device on a linear accelerator platform (cone beam computed tomography, CBCT) [20], management of breathing movements during simulation [21], treatment alone (deep inspiration breath hold, DIBH), rapid and accurate dose administration (volumetric modulated arc therapy, VMAT) [22], the use of high-dose-rate beams without homogenization filters (flattening filter free, FFF) [23], or the possibility of correcting and optimizing the patient’s position by employing the irradiation table with six degrees of freedom [24].

## 2. SBRT

SBRT is still a relatively new radiotherapy technique that can deliver higher doses of radiation to the tumor in only a few treatment fractions. It has been established in the treatment of various solid tumors, such as non-small cell lung cancer, prostate cancer, hepatic cancer, and oligometastatic disease. Emerging evidence suggests that SBRT may play a role in the treatment of PC. It has attracted significant interest for pancreatic cancer patients, as it is completed quickly over one to five fractions, requires less time away from full doses of chemotherapy, and is generally well-tolerated than conventional radiotherapy due to the use of smaller target volumes. Much of the recent data on the use of SBRT in pancreatic cancer reflect delayed local progression in locally advanced cases and improved rates of R0 resection and local control when used in neoadjuvant settings [25,26,27].

### 2.1. Potentially Curative Effect of SBRT

Initial studies [28,29,30] of the low-dose SBRT approach reported equivalent local control rates to those achieved by chemoradiation. These studies prompted efforts to determine whether dose escalation could affect local control (LC) or overall survival (OS). The first phase I dose-escalating study from Stanford in 2009 increased a single dose of radiation in patients with LAPC [31]. The study was terminated at a dose of 25 Gy because 100% local control was achieved in all six evaluable patients treated with this dose. The median survival was 11 months. Despite smaller margins and lower acute toxicity, patients treated on the same single-fraction protocol suffered from a high degree of late toxicity (25% grade ≥ 2) [32]. The authors reported gastric ulcer, duodenal and biliary stricture, and small bowel perforation as sites of late toxicity. Distant failure occurred as the first site of failure.

Using more fractions (usually five), researchers have reported lower levels of toxicity without a reduction in local control. Herman et al. [33] treated their patients with neoadjuvant gemcitabine and SBRT at a dose of 33 Gy in five fractions. The median OS was 13.9 months, and LC at 1 year was 78%. Four patients (8%) underwent margin-negative and lymph-node-negative surgical resections. Park et al. [34] retrospectively compared the same SBRT dose with the conventional approach (45–56 Gy in 25–28 fractions with concurrent chemotherapy). SBRT achieved similar disease control outcomes as IMRT but with less toxicity. In another study conducted by Ryal et al. [35], the median OS was 13 months from diagnosis, and 6- and 12-month LC rates were 91% and 78%, respectively. Patients receiving induction chemotherapy had superior survival from diagnosis to that of patients who did not (14 vs. 7 months, *p* = 0.01). Higher doses of 40 Gy (range 30–50 Gy) in five fractions were used in a retrospective study by Shen et al. [36] SBRT was combined with gemcitabine plus capecitabine chemotherapy. The median OS and progression-free survival (PFS) from the date of diagnosis were 19 and 12 months, respectively. The 1-year and 2-year survival rates were 82.1% and 35.7%, respectively, whereas the 1-year and 2-year PFS rates were 48.2% and 14.3%, respectively. In general, the median survival in these studies is 14–15 months, the annual local control is about 80%, and the grade 3 toxicity is below 10% [37].

In recent years, some nonmatched studies [34,38] directly compared SBRT and conventionally fractionated radiation therapy (CRT), reporting no significant differences in terms of outcomes. However, de Geus et al. and Zhong et al. [39,40] compared matched cohorts treated with SBRT and CRT, reporting improved median OS in the SBRT patient group. Similar results were reported in a meta-analysis by Tchelebi et al. [41]. They included 9 studies on SBRT and 11 studies on CRT in LAPC (1147 patients). For SBRT, the median dose was 30 Gy, and the most common regimen was 30 Gy in five fractions. For CRT, the majority of studies delivered 50.4 Gy in 28 fractions with concurrent gemcitabine. The SBRT technique achieved significantly superior outcomes in terms of 2-year OS (26.9% for SBRT versus 13.7% for CRT, *p* = 0.004). The authors also showed a significantly higher grade 3–4 acute toxicity in patients treated with standard radiotherapy (5.6% for SBRT versus 37.7% for CRT, *p* = 0.013), whereas no differences between the two treatments were recorded in terms of late toxicity.

Recent studies have also shown that LAPC patients treated with aggressive induction chemotherapy in combination with SBRT are more likely to ultimately undergo resection. Moningi et al. [42] evaluated 88 patients treated with SBRT using gemcitabine regimens or FOLFIRINOX. SBRT doses ranged from 25 to 33 Gy in five fractions. The one-year local control rate was 61%, and the median overall survival was 18 months. A proportion of 20% of these LAPC patients underwent surgery. Compared to non-resected patients, those who underwent surgery had a significantly higher median OS (20.2 months vs. 12.3 months, respectively). Grade 3 toxicity was less than 6%. A large institutional study conducted by Mellon et al. [43] treated 159 patients (110 BRPC, 49 LAPC). The authors reported a 24% surgical conversion rate in patients with LAPC treated with FOLFIRINOX chemotherapy and SBRT. All these patients achieved resection with microscopic negative margins despite having “unresectable” disease at diagnosis. Grade 3 or higher toxicity was 7%. The median OS was 34.2 months (nearly 3 years) in patients who underwent resection and 11.3 months in those who did not. Recently, the safety and efficacy of multiagent chemotherapy (FOLFIRINOX or gemcitabine + nab-paclitaxel) in combination with SBRT for the treatment of LAPC was confirmed by Hill et al. in a prospective non-randomized controlled phase II trial [44]. Median OS was 20.2/14.6 months from diagnosis/SBRT, and 1- and 2-year OS from SBRT was 58% and 28%, respectively. A proportion of 39% of patients were surgically explored, and 75% achieved R0 resection. The median OS after resection was 28.6/22.4 months from diagnosis/SBRT, respectively. Only one patient (2.1%) had late-grade ≥ 2 gastrointestinal toxicity attributable to SBRT.

Some studies also suggest the benefit of SBRT for the treatment of patients with a borderline resectable pancreatic tumor. Chuong et al. [45] treated 73 patients (57 BRPC, 16 LAPC) who received an induction combination of gemcitabine, docetaxel, and capecitabine followed by SBRT. The prescribed dose of 30 Gy was increased to 35 Gy in the area of large-vessel infiltration using the simultaneous integrated boost (SIB) technique to ensure maximum probability of tumor regression and R0 resection. After restaging, 56.1% of patients with BRPC underwent surgical resection, with the majority (96.9%) having negative margins. Resected patients had significantly better median OS (19.3 vs. 12.3 months; *p* = 0.03) and median PFS (12.7 vs. 5 months; *p* < 0.0001). No grade 3 acute toxicity was reported. In a subsequent study [43], the same authors treated 159 patients (110 BRPC, 49 LAPC) with neoadjuvant chemotherapy and SBRT. Compared to the study described above, patients received a more aggressive combination of chemotherapy (43% of LAPC patients were administered the FOLFIRINOX regimen); the dose was increased to 30 Gy to the tumor and 40 Gy dose painted to tumor-vessel interfaces. Surgical resection was performed in 51% of patients with BRPC, and R0 resection was achieved in 96% of these patients. Again, the median OS was significantly higher in patients who underwent surgery compared to those who did not (34.2 vs. 14.0 months; *p* < 0.001). Although the prescribed doses were higher than in the previous publication, the incidence of late toxicity was the same (~5%). The feasibility of using SBRT for the treatment of BRPC is supported by the results of other studies [46,47,48], some with a smaller number of enrolled patients. Based on these results indicating improved LC, a higher rate of R0 resectability, and improved OS, SBRT should be recommended in such cases.

The use of local therapy in operable patients is still controversial. Some studies have reported an impact of positive resection margin on survival, whereas others found no such correlation [49]. Cloyd et al. [50] observed that adding preoperative CRT in comparison with systemic therapy alone was associated with a higher rate of margin-negative resection (91% vs. 79%, *p* < 0.01), a lower rate of positive lymph nodes (53% vs. 23%, *p* < 0.01), greater treatment effect, and reduced incidence of locoregional recurrence (LR; 16% vs. 33%, *p* < 0.01) but similar median OS (33.6 vs. 26.4-months, *p* = 0.09). Finalized studies using SBRT for the treatment of pancreatic cancer are listed in Table 1.

### 2.2. Pain Relief

Pancreatic cancer is an aggressive disease caused by its local ground with significant invasion of the surrounding structures [55]. This leads to the activation of sympathetic and parasympathetic nerve fibers and, consequently, to pain [56]. Approximately 30–40% of patients report pain as the dominant symptom at diagnosis, with as many as 90% reporting pain as the dominant symptom before death [57]. Pain relief can be achieved with CRT or SBRT, and studies [33,56,58] suggest that SBRT has a better effect and lower toxicity than standard CRT. SBRT may lead to permanent pain relief in as many as two-thirds of symptomatic patients and can be well-integrated with other therapeutic options, especially chemotherapy.

### 2.3. Oligometastases

SBRT is also widely used to treat oligometastatic disease. Treatment of liver metastases with SBRT has shown promising results with a high degree of local control in other primary malignancies, such as colorectal cancer [29,30,31,32,33,34,35,36,37,38,39,40,41,42,43,44,45,46,47,48,49,50,51,52,53,54,55,56,57,58,59,60,61]. Data on the SBRT treatment of liver metastases from PC are limited but provide promising results. Oladeru et al. [62] reported the results of 41 patients with metastatic pancreatic cancer whose liver metastases were treated with SBRT to a mean dose of 50.0 Gy in five to six fractions. After 12 months, the local control rate was 75.8%, and overall survival was 36.3%. There may also be clinical situations in which SBRT of primary pancreatic lesions may be considered in patients with limited metastases, such as a patient with an oligometastatic disease that has been stable for more than six months or has responded well to systemic therapy [63].

## 3. Practical Considerations

### 3.1. Patient Selection

Pancreatic SBRT is currently reserved for patients with LAPC and BRPC. The indication of SBRT must be based on the assessment of the extend of the disease by a radiation oncologist concerning the overall size of the infiltration and its relationship to the surrounding radiosensitive structures. SBRT is not intended for patients with metastatic disease (with the exception of special cases of oligometastatic disease) or with large tumors or multiple affected lymphatic nodules in the vicinity. Gastrointestinal mucosal infiltration evident at the time of diagnostic endoscopy or endosonography is also a contraindicated due to the direct disruption of wall integrity and the risk of bleeding and peritonitis. The current version of the NCCN guidelines (1.2022) [64] for advanced, inoperable, and non-metastatic pancreatic cancer lists SBRT as a treatment option after induction chemotherapy and as a method equivalent to chemoradiotherapy. If patients are unable to receive chemotherapy, SBRT can also be used as a stand-alone modality.

### 3.2. Neoadjuvant Systemic Therapy

As previously mentioned, optimal management of patients with LAPC and BRPC includes systemic neoadjuvant chemotherapy followed by chemoradiotherapy or SBRT and subsequent resection (if possible). Neoadjuvant chemotherapy is used to downsize the tumor, increase the chance of resectability, test the sensitivity to chemotherapy, and exclude patients with rapidly progressive or hidden metastatic disease who would not benefit from the addition of surgery or radiotherapy. Unfortunately, there is still a lack of randomized data in this setting. In the case of LAPC, it is recommended to start with the most active combined chemotherapy regimen for the treatment of metastatic disease. Preferably modified FOLFIRINOX or gemcitabine plus or minus nab-paclitaxel have been used [65]. Close monitoring of chemotherapy efficacy every 2–3 months is crucial. In the case of regression, resection should be reconsidered. If progression or metastatic spread occurs, there is a need to change the cytostatic regimen and continue with systemic treatment, but the prognosis is usually poor. In the case of minor regression or disease stabilization, radiotherapy should be considered to increase local disease control. In BRPC, there is no consensus regarding the chemotherapy regimen, length of the treatment, role of radiotherapy, and its timing and treatment schedule. Although some data regarding gemcitabine efficacy in this setting have been published [65,66], more effective chemotherapy regimens similar to the LAPC setting are recommended, usually of at least 4 months. The role of SBRT remains controversial [67]. Selected studies evaluating the treatment of BRPC are summarized in Table 2.

### 3.3. Fiducial Placement

**Figure 1:**
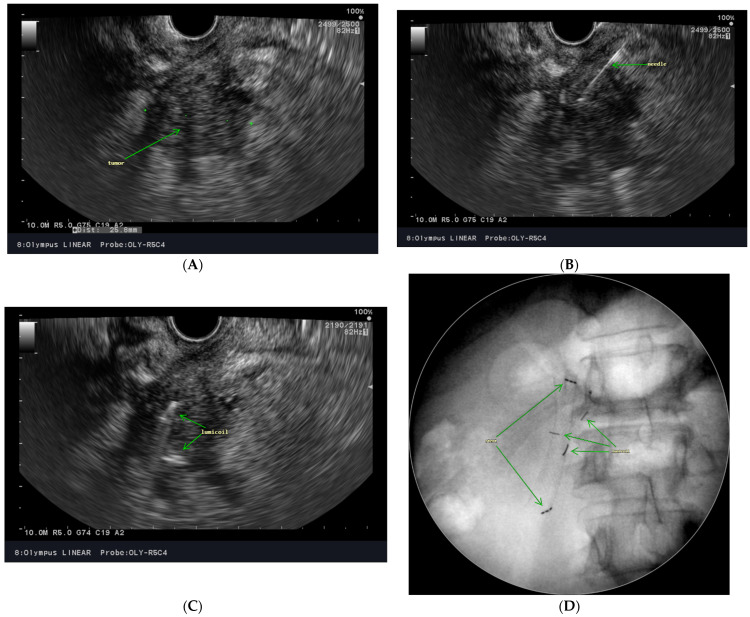
Endoscopic ultrasound-guided fiducial placement. Visualization of the tumor (**A**), needle trajectory (**B**), fiducial insertion (**C**), and control X-ray scan (**D**).

Most trials [33,69,70,71,72] published to date used fiducial markers to localize the target volume during irradiation. In our practice, we insert one to three LumiCoil clips under EUS guidance. The fiducials are placed via a fine needle with a thickness of 19G directly into the tumor, into its periphery, or max. 1 cm from the tumor into the healthy pancreas. To ensure the healing of a possible inflammatory reaction, it is advisable to insert markers at least 2 days before the simulation [73,74]. Biliary stents are not suitable for localization. On the other hand, localization of target volume on daily non-contrast CBCT is more accurate with a biliary stent than with no such surrogate marker of tumor position [75,76]. Figure 1 summarizes a clinical example of endoscopic ultrasound-guided fiducial placement before SBRT.

### 3.4. Planning CT Simulation

A comfortable, accurate, and reproducible position can be ensured using individually prepared shaped vacuum bags, which are attached to a stereotactic frame or used freely. In our practice, we use CIVCO frameless fixation and positioning systems as a standard for patient fixation. We treat with the most used approach, i.e., patients in a supine position with their arms above their heads and their knees supported.

SBRT planning is based on CT examination or its fusion with other 3D examination methods (MR, positron emission tomography (PET), etc.) This fusion ensures both accurate determination of the target volume and accurate planning of irradiation technology. Scanning of one-millimeter sections may be recommended; the examined area must include at least the target lesion and the entire volumes of risk tissues and organs in the vicinity.

Patients must be fasted for at least 3 h before planning CT examination and before each radiotherapy session. During the acquisition of planning CT scans, oral, and intravenous contrast are standardly combined to more accurately locate the tumor site and differentiate between vascular structures, intestines, and nodes. In terms of timing, the combination of arterial (25–35 s after, i.e., contrast application) and venous (55–70 s) phases is ideal. Most authors recommend scanning in the pancreatic parenchymal phase, i.e., 45–50 s after application of the contrast agent [77].

During SBRT treatment planning, it is necessary to use techniques that will help solve the movement of the target volume and organs at risk (OAR) during respiration. It is generally recommended to use breath holding for simulation and subsequent irradiation in the exhalation phase or maximum breath hold (DIBH). Breath holding minimizes the size of the target volume compared to free breathing. Irradiation in the end-expiratory phase of the respiratory cycle is more reproducible than maximum inspiration, but it is necessary to scan more than once. In addition, it is not suitable for some patients [78,79,80]. In our practice, we use the real-time position management (RPM) system developed by Varian to obtain deep-inspiration breath hold. If the patient is unable to hold his breath, 4D-CT technology can be used to scan the patient during normal breathing. The target volumes are then contoured in individual breath phases and fused to form ITV (internal target volume). In such circumstances, epigastric compression, gating, tracking, or their mutual combination is used to minimize diaphragm movements and subsequent tumor movements.

### 3.5. Contouring

We delineate gross tumor volume (GTV) with the assistance of a radiologist utilizing all available information, including CT, MR, PET, and endoscopy reports. According to the recommendation of Oar et al., we use tumor–vessel interface volume (TVI) as a part of the clinical target volume (CTV), together with GTV [26,43,81]. The TVI is the area nearby between major vessels and the GTV; it is important because recurrences or positive surgical margins often remain in this area. Any major vessel within 5 mm of the tumor should be contoured from 5 mm proximal to 5 mm distal of the GTV. The majority of studies have utilized a 5 mm expansion to planning target volume (PTV) [82].

All at-risk organs with an additional safety margin must be excluded from high-dose PTV. To this end, we draw three GI organs, namely the stomach with the duodenum and the small and large intestines. Then, 3–5 mm is added as a safety margin to create the corresponding planning organ at-risk volumes (planning risk volumes, PRV). The sufficient distance between the high dose in PTV and these risk organs is 5–7 mm. The risk is also related to the volume of the PTV and the OAR border; therefore, volume parameters are also included in the constraints [83].

### 3.6. Dose and Fractionation

Published SBRT studies have used one to six fractions, with five fraction schemes being most commonly used [69,84,85]. Ablative dose regimens with 25 Gy in one fraction or 45 Gy in three fractions have shown higher toxicity. Doses up to 50 Gy in five fractions to the PTV have been used in several studies [58,69,86] with acceptable toxicity. In our daily clinical practice, we often follow the recommendations of Oar et al. [26] and prescribe a dose of 40 Gy in five fractions to as much of the PTV as possible. Owing to the vicinity of GI structures, a compromise in dose coverage is needed. We deliver the dose maximum four times a week with no more than two consecutive fractions.

Some authors use intensity-modulated radiotherapy (IMRT) with SIB dose painting, typically with two or three planning target volumes (a dose to get the microscopic disease, an SIB, to the GTV and, if possible, a second SIB at a higher dose to the hypoxic center) [87,88]. This technique with a high dose to the tumor and lower doses to a margin surrounding the gross disease increases the rate and durability of local control.

The need for homogeneous coverage of the target volume with the prescribed dose, as required in CRT due to large volumes containing risk structures, has been abandoned in the SBRT concept. Conversely, allowing a hotspot can improve the conformity of high-dose distribution and thus allow for dose escalation. The tumor center is typically more hypoxic than the periphery and therefore more radioresistant. Thus, a hotspot in the more radioresistant part of the tumor is a benefit.

**Figure 2:**
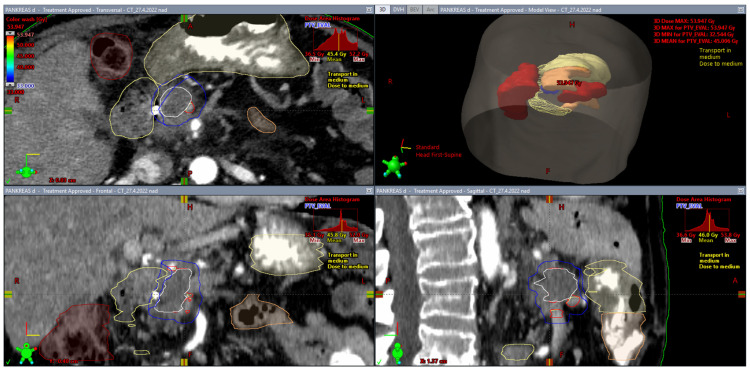
Planning volumes and risk structures. The tumor (GTV, white) and nearby vessels (TVI, red) are contoured. The sum of GTV and TVI forms CTV. A 5 mm expansion of the CTV makes planning target volume (PTV, blue). The main OARs are the duodenum (yellow), stomach (yellow), small bowel (light brown), and large bowel (dark brown). The evaluable PTV (PTV_EVAL, smaller blue volume) is PTV less GI structures with 3–5 mm margins. The fiducial marker is shown at the border of the GTV.

**Figure 3:**
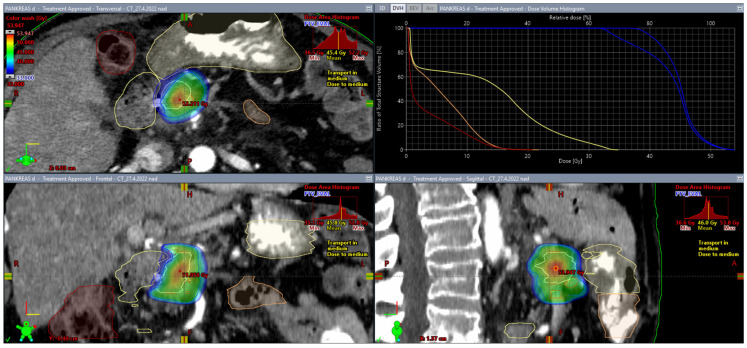
Dose coverage. PTV 38.03 cc (blue; near-maximum dose of 51.26 Gy; mean dose of 43.42 Gy; near-minimum dose of 32.9 Gy), PTV_EVAL 32.01 cc (blue; near-maximum dose of 51.49 Gy; mean dose of 45.01 Gy; near-minimum dose of 38.73 Gy), duodenum (yellow; maximum dose of 32.55 Gy), stomach (yellow; maximum dose of 21.50 Gy), small bowel (orange; maximum dose of 15.11 Gy), and large bowel (brown; maximum dose of 14.88 Gy). The compromise in dose coverage caused by the vicinity of the duodenum and stomach is displayed. The corresponding DVH is plotted in the upper-right corner. Near-maximum and near-minimum doses correspond to ICRU report 83 recommendations.

From this point of view, the SIB approach described above represents homogeneous irradiation of multiple target volumes with gradually increasing doses, i.e., a certain transition between conventional homogeneous irradiation and dose escalation using controlled hotspots within one PTV. The SBRT approach requires a prespecified percentage coverage of the target volume with the prescribed dose. It is recommended that the dose of 90% of an evaluable PTV (PTV less the gastrointestinal PRV) be greater than 100% of the prescription dose [26]. On the contrary, if the minimum dose covering 90% of the PTV is less than 90% of the prescription dose, reduced-dose SBRT or another approach should be considered. Maximum doses of 33 Gy in five fractions to the duodenum and small bowel are associated with a low incidence of toxicity [43,89]. Figure 2 and Figure 3 show a clinical example of a 75-year-old man with LAPC (ductal adenocarcinoma, grade 3, classification T4N0M0) treated with surgery (choledocho-duodeno anastomosis), chemotherapy (FOLFIRINOX, 6 cycles), fiducial placement (LumiCoil), and stereotactic radiotherapy (SBRT, 40 Gy/5 fractions).

### 3.7. Dose Application and Constraints

As mentioned above, when planning and delivering a dose, we use all the technical options that are available for treatment with a linear accelerator in combination with a CT device.

The dose is calculated in the Varian Eclipse planning system with the Acuros XB (AXB) calculation algorithm and correction for heterogeneity. We use a photon beam of two compatible Varian TrueBeam STX ver. 2.7 linear accelerators to apply the dose. To accelerate dose application, these devices use radiation beams without homogenizing filters (FFF, Flattening Filter Free Beams) with high dose rates (1400 MU/minute for a 6 MV FFF beam and 2400 MU/minute for a 10 MV FFF beam) [23]. VMAT (volumetric arc therapy) technology also increases time savings. A typical treatment plan consists of three partial VMAT arcs [22].

Daily correction of the patient’s position before the actual irradiation is performed directly on the radiation table using an integrated imaging system (CBCT, CT with a conical beam of radiation) combined with a radiation table with six degrees of freedom (Perfect Pitch 6-DoF Couch by Varian). The CBCT image can be combined with kV images showing the established localization clips. If their position goes outside the set limits during the treatment, the radiation beam is automatically stopped, and the localization process is repeated [20].

To ensure radiation safety, each plan is verified before the treatment by gamma analysis according to a quality assurance (QA) protocol. The transit dose is also measured during the treatment.

When planning, one must distinguish areas where the application of high doses is relatively safe and where it is necessary not to exceed the constraints and reduce the dose. Strictly respected OAR limits ensure maximum safety and tolerance of treatment without the risk of significant side effects. The organs at risk in pancreatic SBRT are the stomach, duodenum, small and large bowel, spinal cord, liver, and kidneys. OARs must be contoured so that the dose can be evaluated using dose–volume histograms (DVH). In our daily clinical practice we use constraints published by Oar et al. in 2020 [26].

### 3.8. MR Linac-Based Approach

Another way to overcome the limitations caused by GI tract movements is daily online adaptive radiotherapy using new linear accelerators combined with magnetic resonance imaging (MR-linacs). This technology allows for direct visualization of the tumor and critical GI structures during irradiation, as well as allows the possibility of adapting the irradiation plan every day according to the current location of the tumor and the risk structures in the vicinity. Patients can be treated with guided breath hold or with gating on free breathing [90].

Rudra et al. [91] evaluated outcomes of inoperable pancreatic cancer patients treated using adaptive magnetic resonance imaging-guided radiation therapy (MRgRT) with and without dose escalation. There was a significant overall survival benefit of 71% at 2 years in the escalated and daily adapted arm versus 25% in the standard therapy patients. In addition, there was no grade 3 or higher toxicity in the escalated group, whereas three patients in the standard, non-adaptive group had grade 3 or higher toxicity. We are now waiting for the results of a prospective phase II multi-institution study (NCT03621644) evaluating 50 Gy in five fractions using MRgRT technology with daily plan adaptation.

This technology provides a platform for dose escalation in radiotherapy of pancreatic tumors without increasing the number of fractions.

## 4. Discussion

We do not intend for this article to be a meta-analysis or systematic review but seek to provide the reader with a basic overview of the current evidence on the indications for SBRT, with a focus on practical considerations, to assist in the development of SBRT planning and performing irradiation. Several topics remain to be discussed.

### 4.1. Dose and Toxicity

Hypofractionated stereotactic body radiation therapy (SBRT) with a high dose per fraction offers a more effective treatment with the potential to overcome radioresistance in pancreatic cancer, thereby improving local control. In addition, due to its short duration, SBRT favors sequential combination with chemotherapy. However, randomized evidence on dosing and fractionation is still lacking. Recent data suggest improved outcomes if higher radiation doses can be safely administered, although radiation tolerance of nearby stomach and bowel structures remains limiting.

As noted above, some authors using 25 Gy in a single fraction or 45 Gy in three fractions regimens have demonstrated higher toxicity in institutional studies. In a study by Pollom et al. [69], the 6-month and 12-month cumulative incidence of grade ≥ 3 gastrointestinal toxicity was 8.1% and 12.3% in the single fraction group and 5.6% in the multiple fraction group, respectively. There were also significantly fewer cases of grade ≥ 2 toxicity in the multifraction SBRT group (*p* = 0.005). Described toxicities included duodenal perforation, gastric or duodenal ulcer, sclerosis of the gallbladder, duodenal stricture, and upper gastrointestinal bleeding. Hoyer et al. [84] reported that after SBRT with a dose of 45 Gy in three fractions, 64% of patients had grade > 2 toxicity, including nausea and pain, and 23% developed severe mucositis or ulceration of the stomach or duodenum, including one patient who required surgery for perforation.

In contrast, doses up to 50 Gy in five fractions per PTV have been used in a number of studies with acceptable toxicity. For example, Pollom et al. [69] used a median dose of 33 Gy (range 25–45 Gy) in five fractions in the multifraction arm; Song et al. [54] used 45 Gy (35–50 Gy) in five fractions (3–8 fractions); Lin et al. [58] prescribed doses in the range of 35–45 Gy with a daily fraction of 7–9 Gy; and Su et al. [86] administered a dose of 30–36 Gy in three fractions or 40–48 Gy in four fractions. No severe grade 3 toxicity was observed in these studies.

Arcelli et al. (2020) [92] aimed to compare two cohorts of LAPC patients treated with SBRT + CHT vs. CRT + CHT in terms of LC, PFS, OS, and toxicity. No statistically significant differences in terms of acute and late toxicity, PFS, or OS were noted between the two cohorts. Only one case (2.5%) of gastrointestinal bleeding was reported 9 months after SBRT. This study showed no significant differences between SBRT and CRT in terms of OS. This difference could be due to the relatively low BED (biological equivalent dose) administered in the SBRT cohort.

In addition to 90-day perioperative toxicity, a recent study by Hill et al. [93] describes long-term outcomes in a group of patients who underwent subsequent surgical resection after receiving neoadjuvant chemotherapy alone or neoadjuvant chemotherapy followed by SBRT. Doses of 33 Gy (10–15% heterogeneity allowed) were administered in five fractions on five consecutive working days. Negative margins (*p* = 0.001), negative nodules (*p* = 0.001), and pathological complete response (*p* = 0.02) were more frequently achieved with the SBRT regimen. Perioperative morbidity of grade 3 or higher was not significantly different between the two cohorts (*p* = 0.81).

### 4.2. Pain Relief

Patients with locally advanced ductal adenocarcinoma of the pancreas often experience severe pain. Pain relief can be achieved with CRT or SBRT, and studies [33,56,57] suggest that SBRT has a better effect and lower toxicity than standard CRT.

A recent systematic review by Buwenge et al. (2020) [94] analyzed 19 papers reporting pain relief after SBRT. The rate of analgesic reduction or discontinuation ranged between 40 and 100% (median 60.3%) in six studies. The pooled rate was 71.5%. The rate of partial plus complete pain response ranged between 44.4 and 100% (median 78.6%) in nine studies. The pooled rate was 78.3%. The analysis also showed a highly significant positive effect of higher EQD2 (equivalent dose at 2 Gy per fraction) on pain relief. In contrast, the only study in which cases of gastric perforation were reported was that in which the highest doses of SBRT were administered. The result of this analysis indicates that for palliative intent, very high doses of SBRT may have a detrimental effect, whereas intermediate doses appear to be more effective than low doses.

### 4.3. MR Linac-Based Radiotherapy

The studies described above suggest that SBRT is associated with improved clinical efficacy and toxicity profiles compared to conventional radiotherapy techniques. Further dose escalation to the tumor is limited by poor soft tissue visualization on computed tomography imaging during radiation planning and treatment delivery. Magnetic resonance guided tomography (MRgRT) techniques [95] have been introduced to improve the quality of imaging and allow for treatment plan adaptation and reoptimization prior to administration of each fraction. Stereotactic MR-guided adaptive radiotherapy (SMART) is a technique combining X-ray beam delivery, daily adaptive treatment planning, and gating/tracking capability using continuous cine MR images. It is expected to constitute the gold standard for future treatment of not only pancreatic cancer but also cancer at many other sites [96,97].

In a non-randomized study, Heerkens et al. [98] evaluated the feasibility of MR guidance with SBRT as safe, with no cases of grade ≥ 3 acute or late toxicity. Rudra et al. [99] investigated the use of MRgRT with standard and high-dose SBRT and demonstrated that dose escalation in PDAC treatment is feasible. Patients treated with the high dose (40–52 Gy) had a significantly higher survival rate than those in the standard-dose group (30–35 Gy). There was no incidence of severe toxicity in the higher-dose group, with all cases of grade ≥ 3 gastrointestinal toxicity reported in the standard-dose group. A study by Luterstein et al. [100] yielded similar results. A patient with clinical stage III LAPC was irradiated with a high BED dose of 72 Gy after chemotherapy and achieved LC 16 months after irradiation without significant side effects or toxicity. In addition, a multi-institutional study by the American Society for Radiation Oncology suggested that adaptive regimens that allow for safe delivery of BED > 70 Gy may achieve higher OS rates than BED < 70 Gy without increasing toxicity [91].

A recent prospective study by Michalet et al. [101] demonstrated the dosimetric benefit of MR-guided radiotherapy for pancreatic tumors. Thirty patients were treated with a mean dose of 50 Gy. No patient experienced grade > 2 acute toxicity. The most common grade 1–2 toxicities were asthenia, abdominal pain, and nausea. With a median follow-up of 9.7 months, the median OS, 6-month OS, and 1-year OS were 89%, 75%, and 75%, respectively. SMART treatment for pancreatic cancer is feasible without limiting toxicity. Daily adaptation demonstrated benefit in terms of tumor coverage and OAR savings.

## 5. Conclusions

New evidence suggests that SBRT plays an important role in the treatment of PC. Unlike conventional radiotherapy, which has no potential to further improve the overall survival of patients with localized pancreatic cancer, SBRT offers several benefits, including higher BED (biological equivalent dose), reduced volume of irradiated healthy tissue, and shortened overall treatment time. Studies published to date show that the combination of SBRT with new chemotherapy regimens has a significant potential to shift patient survival from months to years. The next step will probably be the widespread use of linear accelerators in combination with magnetic resonance—MR-linacs.

Approximately half of all patients with PC will subsequently experience locoregional recurrence. Clinical trials exploring new treatment paradigms for PC including stereotactic body radiation therapy are recommended by international consensus guidelines. Ongoing studies using SBRT in the therapy of pancreatic cancer are listed in Table 3.

## Figures and Tables

**Table 1:** Finalized studies using SBRT for treatment of pancreatic cancer.

Study, Year	Median Age (Range)	SBRT Dose (Gy)	Fractions	Induction Chemotherapy	Stage	Patients	Median Follow-up (Range)	Conversion Rate	Local Control (%)	Median OS (Months)	1-Year OS (%)	Median PFS (Months)	1-Year PFS (%)	Toxicity
Jung et al., 2019 [27]	64 (38–84)	28 (24–36)	4	FOLFIRINOX/GE based	LAPC	95	15 (2–49)	7.4%	N/A	16.7	67.4	10.2	42.9	3.2% acute G3+ 3.2% late G3+
Herman et al., 2015 [33]	67 (35–87)	33	5	GE	LAPC	49	13.9 (3.9–45.2)	8.0%	78 1-year	13.9	59.2	7.8	32.7	2% acute G2+ 12% late G2+
Park et al., 2017 [34]	68.3 (45–90)	30–33	5	FOLFIRINOX/GE based/FOLFOX	LAPC	44	12.9 (1.7–107.6)	7%	N/A	15.7	56.2	N/A	N/A	0% G3+
Ryan et al., 2018 [35]	74 (68–79)	28 (25–33)	5	FOLFIRINOX/GE/GE based	LAPC	29	15 (4–18)	N/A	78 1-year	13	51.7	6	17.2	10% acute G3+ 4% late G3+
Shen et al., 2019 [36]	62 (38–84)	40 (30–50)	5	GE+capecitabine	LAPC	56	17 (3–43)	N/A	N/A	19	82.1	12	48.2	3.6% acute G3 5.4% late G3 3.6% late G4
Mellon et al., 2015 [43]	66.5 (45–85)	median 30–40	5	FOLFIRINOX/GTX/GE based	LAPC	49	14 (4–46)	N/A	N/A	15	46.9	13.2	34.7	7% acute & late G3+
					BRPC	110		51.0%		19.2	63.6	11.9	43.6	
Hill et al., 2022 [44]	N/A	N/A	5	FOLFIRINOX/GE + nab-paclitaxel	LAPC	48	60 (14-65)	38.6%	23.9 months (median local PFS)	20.2	58	N/A	N/A	2.1% late G2+
Chuong et al., 2013 [45]	64 (38–87)	35 (25–50)	5	GTX	LAPC	16	11 (2.2–21)	N/A	81 1-year	15	68.1	9.8	41	0% acute G3+ 5.3% late G3+
					BRPC	57	7.8 (3.4–25.9)	56.1%		16.4	72.2	9.7	42.8	
Moningi et al., 2015 [46]	67.2 (35–87)	25–33	5	FOLFIRINOX/GE/GE based	LAPC	74	14.5	21.6%	61% 1-year	18.4	N/A	9.8	N/A	3.4% acute G3+ 5.7% late G2+
					BRPC	14	10.3			14.4				
Mahadevan et al., 2011 [51]	67 (44–88)	24–36	3	GE	LAPC	39	21 (6–36)	N/A	85 crude	20	68.1	15	55.3	9% late G3+
Zhang et al., 2018 [52]	64 (44–80)	30–36	5 or 6	N/A	LAPC	41	12.4 (2.8–24)	N/A	N/A	11.8	46.3	N/A	N/A	N/A
Kim et al., 2019 [53]	74 (56–92)	29 (25–42)	3 or 5	FOLFIRINOX	LAPC	27	9 (3–32.7)	N/A	61 1-year	11.6	40.7	N/A	N/A	22% G3 22% G2 0% G4
Song et al., 2015 [54]	62 (28–86)	45 (35–50)	3–8	N/A	LAPC	59	10.9 (3.2–48.7)	N/A	90.8% 1-year	12.5	53.9	13.9	N/A	1.7% late G3 0% late G4

SBRT = stereotactic body radiotherapy; BRPC = borderline-resectable pancreatic cancer; LAPC = locally advanced pancreatic cancer; Gy = Grays; OS = overall survival; PFS = progression-free survival; G = grade; N/A = not applicable; GTX = gemcitabine + docetaxel + capecitabine; FOLFIRINOX = oxaliplatin + folinic acid + irinotecan + fluorouracil; GE = gemcitabine alone or gemcitabine + nab-paclitaxel; FOLFOX = oxaliplatin, folinic acid, and fluorouracil.

**Table 2:** Selected studies evaluating the treatment of BRPC.

Study, Year	Stage	Therapy	Patients	R0 (%)	Median OS (Months)	HR
Versteine et al., 2021 [65]	BRPC	Surgery → gemcitabine	54	8.5	13.2	HR 0.67 *p* = 0.045
		CRT (gemcitabine) → Surgery → gemcitabine	59	40.7	17.3	
Katz et al., 2021 [66]	BRPC	FOLFIRINOX → Surgery → FOLFOX	70	42	30	NR
		FOLFIRINOX → SBRT → Surgery → FOLFOX	56	25	17.1	
Jang et al., 2018 [67]	BRPC	Surgery	23	26	12	HR 1.495 *p* = 0.028
		CRT → Surgery	27	52	21	
Motoi et al., 2019 [68]	Resectable + BRPC	Surgery → S1	180	72	26.7	HR 0.75 *p* = 0.015
		gemcitabine + S1 → Surgery → S1	182	77	36.7	

BRPC = borderline-resectable pancreatic cancer; CRT = chemoradiotherapy; SBRT = stereotactic body radiotherapy; R0 = number of R0 resections; HR = hazard ratio; NR = not reported; FOLFIRINOX = oxaliplatin, folinic acid, irinotecan, and fluorouracil; FOLFOX = oxaliplatin, folinic acid, and fluorouracil.

**Table 3:** Ongoing studies using SBRT in the therapy of pancreatic cancer.

ClinicalTrials.gov Identifier	Study Title	Study Phase	Patients	Estimated Study Completion Date	Primary Outcome Measures	Secondary Outcome Measures	Study Arms
NCT03563248	A Randomized Phase 2 Study of Losartan and Nivolumab in Combination With FOLFIRINOX and SBRT in Localized Pancreatic Cancer	2	160	31 December 2025	Proportion of participants with R0 resection	Progression-free survival Overall survival Pathologic complete response Number of participants with treatment related serious adverse events	Experimental: FOLFIRINOX + Losartan:SBRT + Losartan:Surgery FOLFIRINOX + Losartan:SBRT + Nivolumab + Losartan:Surgery FOLFIRINOX × 8:SBRT + Nivolumab:Surgery Comparator: FOLFIRINOX: SBRT: Surgery
NCT02128100	The Effect of FOLFIRINOX and Stereotactic Body Radiation Therapy for Locally Advanced, Non-Resectable Pancreatic Cancer	2	28	May 2025	Number of Participants with Adverse Event(s) as a Measure of Safety and Tolerability	Overall Response Rate for Participants	Experimental: FOLFIRINOX with SBRT
NCT04089150	MASTERPLAN: A Randomised Phase II Study of MFOLFIRINOX And Stereotactic Radiotherapy (SBRT) for Pancreatic Cancer With High Risk and Locally Advanced Disease	2	120	30 August 2023	Locoregional control (Locoregional Response Rate LRR)	Safety (NCI CTCAE v5.0), Surgical morbidity/mortality, Radiological response rates, Progression Free Survival, Pathological response rates, Surgical resection rates, R0 resection rates, Quality of Life, Deterioration-Free Survival, Overall survival	Experimental: Option 1: mFOLFIRINOX (6 cycles) Option 2: gemcitabine + nab-paclitaxel (3 cycles) Stereotactic Radiotherapy (SBRT) Comparator: Option 1: mFOLFIRINOX (6 cycles) Option 2: gemcitabine + nab-paclitaxel (3 cycles)
NCT03777462	Comparisons of Different Neoadjuvant Chemotherapy Regimens With or Without Stereotactic Body Radiation Therapy for Borderline Resectable Pancreatic Cancer: Study Protocol of a Prospective, Randomized Phase II Trial	2	150	31 December 2022	Overall time (Time Frame: From date of randomization until the date of death from any cause, whichever came first)	Disease free time (Time Frame: From date of randomization until the date of first documented progression or metastasis)	Experimental: Neoadjuvant gemcitabine plus nab-paclitaxel with SBRT Neoadjuvant S-1 plus nab-paclitaxel with SBRT Comparator: Neoadjuvant gemcitabine plus nab-paclitaxel
NCT04698915	GRECO-2: A Randomized, Phase 2b Study of GC4711 in Combination With Stereotactic Body Radiation Therapy (SBRT) in the Treatment of Unresectable or Borderline Resectable Nonmetastatic Pancreatic Cancer	2	160	October 2027	Median Overall Survival after SBRT completion	Median Progression Free Survival after SBRT Completion per RESIST 1.1	Experimental: Drug GC4711 + SBRT Comparator: Placebo + SBRT
NCT05114213	MR-Guided Adaptive Stereotactic Body Radiotherapy (SBRT) of Primary Tumor for Pain Control in Metastatic Pancreatic Ductal Adenocarcinoma (mPDAC)—a Randomized, Controlled Clinical Study		92	May 2024	Mean cumulative pain index	Number of biliary complications, Malnutrition, Treatment toxicity, Death from any cause	Experimental: Strandard of care chemotherapy + SBRT Comparator: Strandard of care chemotherapy
NCT03492671	A Phase II Trial of Pre-operative Chemotherapy (With Gemcitabine and Nab- Paclitaxel) and Stereotactic Body Radiotherapy Followed by Surgery and Chemotherapy in Patients With Resectable Pancreatic Adenocarcinoma	2	30	30 September 2024	Curative Intent Resection (R0) rate	Disease Free Survival Rate Overall Survival Rate	Experimental: Chemotherapy (Gemcitabine + nab-paclitaxel) and SBRT
NCT05116917	Nivolumab, Ipilimumab and Radiation in Combination With Influenza Vaccine in Patients With Pancreatic Cancer (INFLUENCE)		30	1 December 2024	Objective response rate (ORR)	Duration of response (DoR), Disease control rate (DCR), Progression free survival (PFS), Overall survival (OS), EORTC QLQ-C30, Treatment-related adverse events as assessed by CTCAE v5.0	Experimental: Nivolumab + Ipilimumab + Influenza vaccine + SBRT
NCT04331041	Phase II Study of Stereotactic Body Radiotherapy and Focal Adhesion Kinase Inhibitor in Advanced Pancreas Adenocarcinoma	2	42	31 July 2025	Progression-free survival (PFS)	Safety and toxicity profile of the regimen as measured by incidence of acute and late adverse events, Overall survival, Distant metastasis progression-free survival, Objective response rate	Experimental: MR-guided SBRT + Defactinib Comparator: MR-guided SBRT
NCT03991962	Phase II Study to Evaluate Modified Folfirinox and Stereotactic Body Radiation Therapy in Non-metastatic Unresectable Pancreatic Adenocarcinoma	2	28	1 February 2023	Progression Free Survival	Radiographic Response, Rates of Recurrence, Rates of grade 3 or greater gastrointestinal toxicity, Overall Survival	Experimental: mFOLFIRINOX followed by SBRT
NCT03073785	A Randomized Phase II Study of the Efficacy and Safety of Hypofractionated Stereotactic Radiotherapy and 5FU or Capecitabine With and Without Zometa in Patients With Locally Advanced Pancreatic Adenocarcinoma	2	44	December 2022	Local control at 4, 8 and 12 months	Maximum tolerated dose of zoledronic acid, Local failure-free survival, Overall survival, Surgical complete resection, Pathologic response for patients who undergo resection, The change of tumor size after SBRT, The change of max and average SUV after SBRT, Tumor and organ motion	Experimental: Zoledronic acid, chemotherapy, radiation therapy Comparator: Chemotherapy, radiation therapy
NCT04090463	A Phase II Study of Primary Chemotherapy, Stereotactic Body Radiation Therapy, and Intraoperative Radiation Therapy in Borderline Resectable Pancreatic Adenocarcinoma	2	100	30 December 2027	Disease-specific survival	Progression-free survival, Number of participants with treatment-related adverse events as assessed by CTCAE v4.0, Rate of margin-free surgery, Rate of surgical complications, Resection rate	Experimental: IORT group
NCT04789486	Nano-SMART: An Adaptive Phase I-II Trial of AGuIX Gadolinium-based Nanoparticles With Stereotactic Magnetic Resonance-guided Adaptive Radiation Therapy for Centrally Located Lung Tumors and Locally Advanced Unresectable Pancreatic Ductal Adenocarcinoma	1–2	100	10 September 2024	Maximum tolerated dose (MTD) Phase 1, Compare Local Control at 12 months of Maximum tolerated dose MTD—Phase 2	Progression-free survival (PFS), Overall Response Rate (ORR) at Maximum tolerated dose (MTD), Serious Adverse Events at 90 Days and 12 months, Tumor Changes, Compare disease-specific survival, Compare R0 resection rate, Compare overall survival, Quality of Life (QoL)	Experimental: AGUIX + SMART Phase 1 Experimental: AGUIX + SMART Phase 2 Experimental: SMART Phase 2
NCT04986930	Randomized Phase 2 Study of mFOLFIRINOX With or Without Stereotactic Body Radiotherapy in Patients With Locally Advanced Pancreatic Adenocarcinoma	2	92	14 August 2024	1-year progression-free survival rate	Overall survival, Progression-free survival, Overall response rates, Adverse events, Surgical resection rate	Experimental: SBRT+mFOLFIRINOX Comparator: mFOLFIRINOX
NCT04570943	Phase II Study to Assess the Interest of a Sequential Treatment With Gemcitabine/Nab-paclitaxel (GEMBRAX) and Then FOLFIRINOX Followed by Stereotactic Magnetic Resonance-guided Adaptive Radiotherapy in Patients With Locally Advanced Pancreatic Cancer	2	103	October 2026	Rate of non-progression at 4 months, Acute gastrointestinal non-toxicity rate	Assessment of adverse events due to CHT and RT, Progression-free Survival (PFS), Overall Survival (OS), Resection rate, Healthy margin resection rate (R0), Prognostic impact of CA 19-9 changes on survival, Quality of life, Correlation of PTV coverage and dose received by the GTV with PFS and OS, Correlation of the dose received by organs at risk with the appearance of GI toxicities	Experimental: Gabrinox followed by stereotactic radiotherapy
NCT04390399	Open-label, Randomized, Comparative Phase 2 Study of Combination Immunotherapy Plus Standard-of-care Chemotherapy Versus Standard-of-care Chemotherapy for the Treatment of Locally Advanced or Metastatic Pancreatic Cancer	2	328	30 September 2024	Progression Free Survival (PFS)	Objective response rate (ORR), Complete response (CR) rate, and Disease Control Rate (DCR), Overall Survival (OS), Quality of Life (QoL)	Experimental: SBRT + cyclophosphamide + gemcitabine + nab-paclitaxel + aldoxorubicin HCl + N-803 SBRT + cyclophosphamide + gemcitabine + nab-paclitaxel+ aldoxorubicin HCl + N-803 + PD-L1 t-haNK Comparator: SBRT + gemcitabine + nab-paclitaxel Experimental: SBRT + cyclophosphamide + gemcitabine + nab-paclitaxel+ aldoxorubicin HCl + N-803 + PD-L1 t-haNK SBRT + cyclophosphamide + gemcitabine + nab-paclitaxel + aldoxorubicin + N-803 + PD-L1 t-haNK Comparator: Irinotecan liposome + 5-FU/leucovorin
NCT04247165	LAPTOP: Phase 1/2 Study in Locally Advanced Pancreatic Cancer to Assess Safety and Potential Efficacy of Dual Checkpoint Inhibition in Combination With Gemcitabine and Nab-paclitaxel Followed by Immune-chemoradiation.	1–2	20	February 2024	Incidence of treatment-related AEs, SAEs, AEs leading to discontinuation, death, and laboratory abnormalities	Median PFS, OS, Objective Response Rate (ORR), Rate of downstaging to surgical resection	Experimental: Gemcitabine + Nab-paclitaxel + Nivolumab + Ipilimumab + SBRT
NCT03767582	A Phase I/II Trial of Combination Immunotherapy With Nivolumab and a CCR2/CCR5 Dual Antagonist (BMS-813160) With or Without GVAX Following Chemotherapy and Radiotherapy for Locally Advanced Pancreatic Ductal Adenocarcinomas (PDACs).	1–2	30	March 2023	Number of Participants experiencing study drug-related toxicities, Percentage of participants treated with immunotherapy who achieve an immune response	Overall survival (OS), Metastasis free survival (MFS), Local progression free survival (LPFS), Surgical Resectability Rate, Pathological Response Rate, Change in Quality of life score	Experimental: Phase I GVAX/Nivolumab/CCR2/CCR5 dual antagonist Phase II Arm A: Nivolumab/CCR2/CCR5 dual antagonist Arm B: Nivolumab/GVAX/CCR2/CCR5 dual antagonist

## Data Availability

Not applicable.
